# Insulin-Regulated Trafficking of GLUT4 Requires Ubiquitination

**DOI:** 10.1111/j.1600-0854.2010.01113.x

**Published:** 2010-09-20

**Authors:** Christopher A Lamb, Rebecca K McCann, Jacqueline Stöckli, David E James, Nia J Bryant

**Affiliations:** 1 Henry Wellcome Laboratory of Cell Biology, Institute of Molecular, Cell & Systems Biology, College of Medical, Veterinary and Life Sciences, Davidson Building, University of GlasgowGlasgow G12 8QQ, United Kingdom; 2 Diabetes and Obesity Program, Garvan Institute of Medical Research384 Victoria Street, Darlinghurst, Sydney NSW 2010, Australia

**Keywords:** adipocytes, GLUT4, GLUT4-storage vesicles, insulin-regulated, ubiquitin

## Abstract

A major consequence of insulin binding its receptor on fat and muscle cells is translocation of the facilitative glucose transporter GLUT4 from an intracellular store to the cell surface where it serves to clear glucose from the bloodstream. Sorting of GLUT4 into its insulin-sensitive store requires the GGA [Golgi-localized, γ-ear-containing, ADP ribosylation factor (ARF)-binding] adaptor proteins, but the signal on GLUT4 to direct this sorting step is unknown. Here, we have identified a role for ubiquitination of GLUT4 in this process. We demonstrate that GLUT4 is ubiquitinated in 3T3-L1 adipocytes, and that a ubiquitin-resistant version fails to translocate to the cell surface of these cells in response to insulin. Our data support a model in which ubiquitination acts as a signal for the trafficking of GLUT4 from the endosomal/***trans***-Golgi network (TGN) system into its intracellular storage compartment, from where it is mobilized to the cell surface in response to insulin.

A major action of insulin is to stimulate glucose transport into fat and muscle cells. This tissue-specific process is responsible for the clearance of glucose from the bloodstream, and is mediated via regulated exocytosis of the facilitative glucose transporter GLUT4 (1). Defective insulin-stimulated glucose transport is a principle defect underlying the insulin resistance associated with type 2 diabetes (2). In the absence of insulin, GLUT4 is retained intracellularly in tubulo-vesicular structures associated with the trans-Golgi network (TGN)/endosomal system, from where it is trafficked to the plasma membrane in response to insulin (1). The trafficking itineraries that underpin the biogenesis of these GLUT4 storage vesicles (GSVs; also termed IRVs for insulin-responsive vesicles) are yet to be precisely defined. In particular, the signal(s) that direct GLUT4 into GSVs are not understood.

The GGA [Golgi-localized, 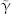-ear-containing, ADP ribosylation factor (ARF)-binding] family of clathrin adaptor proteins plays a role in sorting GLUT4 into GSVs (3,4). Overexpression of a dominant negative GGA mutant in adipocytes inhibits insulin-dependent GLUT4 trafficking, but has no effect on the trafficking of other proteins, such as GLUT1 and the transferrin receptor (TfR), that are not found in GSVs (3). This appears to be because of a block in GSV biogenesis, with the entry of newly synthesized GLUT4 into the insulin-responsive pool being GGA dependent (3,4). The trigger for sorting of GLUT4 into this pathway is, at present, unknown.

The GGAs facilitate delivery of cargo proteins from the TGN into the endosomal system through recognition of attached ubiquitin moieties (5,6). As with other fundamental cellular processes, many aspects of the ubiquitin- and GGA-dependent sorting of proteins from the TGN into the endosomal system are conserved through evolution with similar molecular mechanisms being involved in the analogous pathways that exist in yeast and mammalian cells (6–8).

The yeast *Saccharomyces cerevisiae* is a well-established model system to study membrane traffic in eukaryotes (9). Here, we have utilized this yeast to investigate the role of ubiquitination in GLUT4 traffic. By expressing human GLUT4 in *S. cerevisiae* we demonstrate that it traffics to the endosomal system in a manner that relies on ubiquitin-acceptor sites and GGA proteins. Furthermore, we find that GLUT4 is ubiquitinated both in yeast and in adipocytes, and that a ubiquitin-resistant version mislocalizes under basal conditions. Immunofluorescence microscopy and cell fractionation studies performed in adipocytes indicate that this ubiquitin-resistant version of GLUT4 is not sorted into GSVs and, accordingly, fails to translocate to the cell surface in response to insulin.

## Results

### Trafficking of GLUT4 through yeast endosomes requires ubiquitin-acceptor sites and GGA proteins

**Figure 1:**
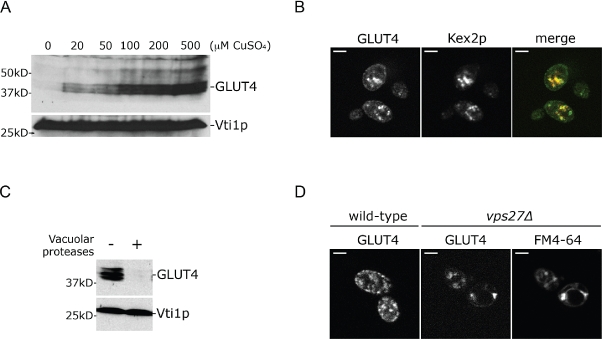
**When expressed in yeast, the insulin-regulated glucose transporter GLUT4 traffics through the endosomal system.** A) Yeast (SF838-9D) harboring a plasmid (pRM2) carrying the human GLUT4 ORF behind the *CUP1* promoter were grown in media (SD-ura) containing increasing concentrations of CuS0_4_. Lysates from these cells were immunoblotted for GLUT4 and Vti1p (as a loading control). B) hGLUT4 (green in the merged image) and HA-tagged Kex2p (red in the merged image) were immunolocalized in yeast cells (SF838-9D) producing both, grown in SD-ura + 100µm CuSO_4_. C) Yeast containing or lacking active vacuolar proteases (RPY10 or SF838-9D, respectively) and producing human GLUT4 (from pRM2) were grown in media (SD-ura) containing 100 µm CuSO_4_. Lysates from these cells were immunoblotted as in (A). D) GFP-tagged hGLUT4 (expressed from pRM34 using 100 µm CuSO_4_) was visualized in wild-type (SF838-9D) yeast and a congenic *vps27*Δ mutant labeled with FM4-64 to mark the class E compartment. Scale bars in (C) and (D) = 2 µm.

Previous studies expressing mammalian GLUT4 in *S. cerevisiae* reported that the transporter localizes to the endoplasmic reticulum (ER) (10,11). We reasoned that this ER retention may be because of the high levels of expression used in these studies, and therefore expressed GLUT4 from the regulatable *CUP1* promoter to achieve lower levels of expression. Expression from the *CUP1* promoter is sensitive to the concentration of Cu^2+^ ions in the growth media, allowing regulation of expression levels (12). Increasing the concentration of CuSO_4_ in the growth media of yeast cells harboring the human *GLUT4* gene under *CUP1* control leads to increased levels of GLUT4 (Figure 1A). When produced at modest levels (using 100 µm CuS0_4_), GLUT4 colocalized with the yeast TGN marker Kex2p (Figure 1B). Although GLUT4 protein could be detected in yeast devoid of active vacuolar proteases because of a *pep4-3* mutation (13) (Figure 1A,B), we were unable to detect the transporter in congenic wild-type cells containing active vacuolar proteases (Figure 1C). Proteins localize to the yeast TGN by continually cycling through the yeast endosomal system including the ‘prevacuolar’ compartment, an exaggerated form of which accumulates in the Class E *vps* mutants such as *vps27*Δ(14–16). This Class E compartment accumulates recycling resident Golgi proteins, biosynthetic vacuolar cargo and endocytosed material (14,15). Consistent with its TGN localization in yeast, GLUT4 becomes trapped in the Class E compartment of *vps27*Δ mutant cells (Figure 1D). GLUT4 appears to be trafficked directly from the TGN to endosomes rather than via the plasma membrane followed by endocytosis because its entry into the class E compartment is not inhibited by latrunculin A which blocks endocytosis (data not shown). The data presented in Figure 1 indicate that, when expressed in yeast, GLUT4 is trafficked to the endosomal system, displaying steady-state localization to the TGN by continually cycling through the endosomal system including compartments containing active vacuolar proteases. This situation is reminiscent of the continual cycling through the adipocyte endosomal system that results in colocalization of GLUT4 with the TGN marker syntaxin-16 (Sx16) under basal conditions (17).

**Figure 2:**
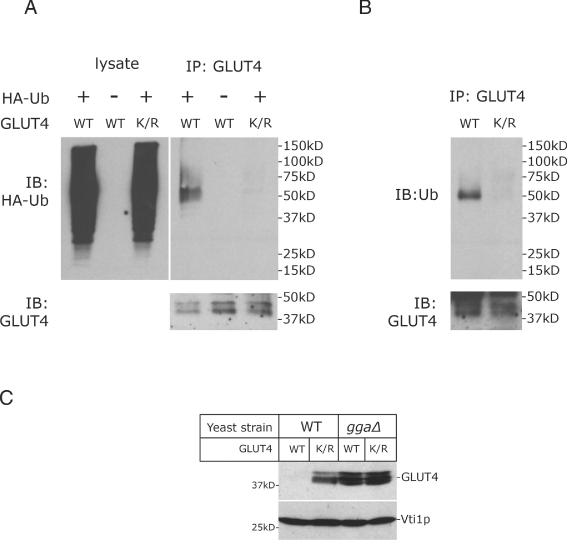
**GLUT4 is ubiquitinated in yeast, and its endosomal trafficking requires both ubiquitin-acceptor sites and GGA proteins.** A) Polyclonal GLUT4 antibodies were used to immunoprecipitate either the wild-type protein (WT; from pRM2) or G4-7K/R (K/R; from pRM3) from yeast lacking active vacuolar proteases (SF838-9D grown in the presence of 100 µm CuSO_4_) that were either coexpressing HA-tagged ubiquitin (HA-Ub from pRM1) or not. Immunoprecipitates, and 1% of the lysates from which the transporter was precipitated, were immunoblotted with monoclonal antibodies that recognize the HA epitope and GLUT4. B) Polyclonal GLUT4 antibodies were used to immunoprecipitate either the WT protein or K/R from yeast (SF838-9D). Immunoprecipitates were immunoblotted with monoclonal antibodies against ubiquitin and GLUT4. C) Lysates prepared from WT or *gga*Δ yeast cells (SEY6210 and MBY004, respectively; both containing active vacuolar proteases) producing either WT GLUT4 (from pRM2) or K/R (from pRM3) were immunoblotted for GLUT4 and Vti1p (as a loading control).

Ubiquitination of membrane protein serves as a signal to direct traffic from the TGN into the endosomal system (6). To investigate whether ubiquitination is involved in the endosomal trafficking of GLUT4, we immunoprecipitated GLUT4 from yeast cells (lacking active vacuolar proteases) and probed for the presence of ubiquitin. Immunoblot analysis of immunoprecipitated GLUT4 with antibodies that specifically recognize ubiquitin (or an epitope-tagged version of ubiquitin) revealed that the transporter is ubiquitinated (Figure 2A,B). Ubiquitin is conjugated to proteins through an amide linkage between its carboxy-terminus and a primary amino group, often contributed by a lysine residue, on the target protein (18). GLUT4 has seven lysines predicted to be cytosolically disposed (19,20). A GLUT4 mutant, with these seven lysines mutated to arginines (G4-7K/R), was expressed in yeast. In contrast to the wild-type protein, the G4-7KR mutant was neither ubiquitinated (Figure 2A,B) nor degraded by vacuolar proteases (Figure 2C), indicating that ubiquitination of GLUT4 is required to target it into the proteolytically active yeast endosomal system. The lack of degradation of the G4-7KR mutant is not because of it being retained in the ER as a result of misfolding. Firstly, the mutant displays a punctate staining pattern by immunofluorescence microscopy (this partially overlaps with Kex2p, but not as well as wild-type GLUT4, perhaps indicating a slightly altered localization), and secondly fusion of a ubiquitin moiety in-frame to the C-terminus of G4-7K/R restores the Pep4p-dependent degradation of the mutant (data not shown).

Ubiquitin-dependent sorting from the TGN into the endosomal system requires the GGA proteins in both yeast and mammalian cells (6,8). Building on our finding that ubiquitination of GLUT4 is required for its delivery to the proteolytically active endosomal system in yeast, we investigated whether the GGA proteins are also involved in this process. To test this we expressed wild-type GLUT4 in mutant yeast cells lacking GGA proteins (*gga*Δ; Figure 2C). It should be noted that these *gga*Δ cells contain active vacuolar proteases. In contrast to the low levels of GLUT4 found in their congenic parent strain, *gga*Δ cells have markedly increased levels of the transporter (Figure 2C), indicating that the GGA proteins are required to deliver GLUT4 to the proteolytically active endosomal system. Steady-state levels of the ubiquitin-defective GLUT4 mutant (G4-7K/R) are similar in wild-type and *gga*Δ cells, and not significantly different to those observed for wild-type GLUT4 in *gga*Δ cells (Figure 2C). These data are consistent with a model in which ubiquitination of heterologously expressed GLUT4 facilitates trafficking of the transporter into the proteolytically active endosomal system in concert with GGA proteins.

### GLUT4 is ubiquitinated in adipocytes

**Figure 3:**
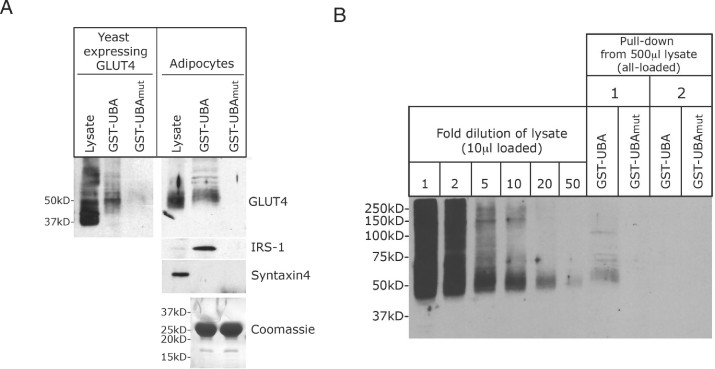
**Endogenous GLUT4 is ubiquitinated in adipocytes.** A) Cell lysates prepared from either 3T3-L1 adipocytes or yeast (SF838-9D) expressing GLUT4 were incubated with 10 µg of either a GST-fusion protein harboring the ubiquitin-binding domain of Dsk2p (GST–UBA) or a mutant version thereof (GST–UBA_mut_) immobilized on glutathione-Sepharose. Following extensive washing, immunoblot analysis was used to determine whether the indicated proteins had bound to these fusion proteins [a sample of the lysates was also included in this analysis; IRS-1 was included as a protein known to be ubiquitinated in adipocytes (22), and Syntaxin4 was included as an abundant protein in adipocytes for which no evidence of ubiquitination exists]. A Coomassie stained gel of the input of the GST fusions is also shown. B) Lysate prepared from four 10-cm plates of 3T3-L1 adipocytes was adjusted to a protein concentration of 5 mg/mL with lysis buffer containing 1 mm NEM. Five hundred microliters of this was incubated with 20 µg of either a GST-fusion protein harboring the ubiquitin-binding domain of Dsk2p (GST–UBA) or a mutant version thereof (GST–UBA_mut_) immobilized on glutathione-Sepharose at 4°C for 2 h with continual mixing. Beads and associated proteins were collected by centrifugation (5 min, 500 ×***g***). The supernatant from this centrifugation step was incubated with another batch of GST-fusion protein (the same as in the first round of binding). Immunoblot analysis was used to determine the amount of GLUT4 that had bound to the indicated fusion proteins during the first (1) and second (2) incubation. To estimate the proportion of total cellular GLUT4 bound to the GST–UBA fusion protein, 10 µL of the lysate, and dilutions thereof (as indicated) were included in this analysis. Ten microliters of lysate diluted 1-, 2-, 5-, 10-, 20- and 50-fold represents 2, 1, 0.4, 0.2, 0.1 and 0.04% of the lysate used in the pull-downs, respectively.

To extend our finding that ubiquitination of heterologously expressed GLUT4 regulates trafficking of the transporter in yeast, we set out to establish whether GLUT4 is subject to ubiquitin-dependent trafficking in adipocytes. We were unable to detect ubiquitinated GLUT4 using an immunoprecipitation approach as used in our yeast studies (Figure 2), and therefore turned to a GST pull-down approach. Like wild-type GLUT4 expressed in yeast, endogenous GLUT4 in adipocytes bound specifically to the ubiquitin-binding UBA (ubiquitin-associated) domain of Dsk2p (21) (Figure 3) as did the insulin receptor substrate IRS-1, which is known to be ubiquitinated in adipocytes (22). In contrast, the abundant adipocyte protein Syntaxin4 (23), for which no evidence of ubiquitination is published, did not bind to the Dsk2p UBA domain (Figure 3). To further control for the specificity of interaction of GLUT4 with the GST–UBA fusion protein, we took advantage of the published structure of the Dsk2p UBA domain in complex with ubiquitin (24) to create a mutant version of the UBA domain predicted to abolish ubiquitin binding (24) (see Appendix S1 for details). Mutation of Met-342 and Phe-344 of Dsk2p abolished binding of GLUT4 and also the IRS-1 [included as a positive control for a protein known to be ubiquitinated in adipocytes (22)] (Figure 3). These data indicate that, as in yeast, GLUT4 is ubiquitinated in adipocytes.

To estimate the proportion of total cellular GLUT4 that is ubiquitinated in 3T3-L1 adipocytes, we used the GST–UBA fusion protein to quantitatively pull down ubiquitinated GLUT4 from a cell lysate. By comparing the amount of GLUT4 pulled down by the GST–UBA fusion with a serial dilution of the original lysate, we estimated that approximately 0.1% of GLUT4 is ubiquitinated in adipocytes (Figure 3B) perhaps indicating that the modification might be a transient one (please see *Discussion*).

### A ubiquitin-resistant version of GLUT4 fails to sort into GSVs and translocate to the cell surface of adipocytes

**Figure 4:**
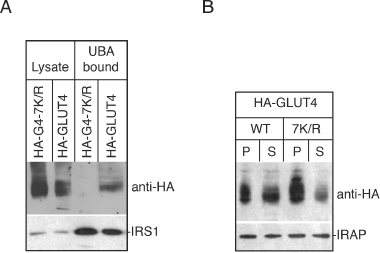
**A ubiquitin-resistant version of GLUT4 displays altered localization in adipocytes.** A) Cell lysates prepared from 3T3-L1 adipocytes expressing either wild-type (WT) HA-GLUT4 or HA-G4-7K/R were incubated with GST–UBA as in Figure 3. Immunoblot analysis was used to determine whether the tagged versions of GLUT4 had bound (anti-HA). This analysis included a sample of the lysates, and the bound material was also probed for the presence of IRS-1 as a ubiquitinated protein known to bind to GST–UBA (Figure 3). B) 3T3-L1 adipocytes expressing either WT HA-GLUT4 or HA-G4-7K/R (7K/R) were homogenized and subjected to 16 000 ×***g*** centrifugation for 20 min. The amount of either WT or 7K/R HA-tagged GLUT4 and IRAP in the pellet (P) and supernatant (S) was determined by immunoblot analysis.

To investigate the role of ubiquitination in insulin-regulated GLUT4 trafficking we expressed the G4-7K/R mutant in adipocytes. In contrast to its wild-type counterpart, a HA-tagged version of this mutant does not bind to the GST–UBA fusion used in Figure 3 (Figure 4A), indicating that it is not ubiquitinated in adipocytes, as predicted from our yeast studies. Our yeast studies demonstrated a role for the GGA family of clathrin adaptor proteins in the ubiquitin-dependent sorting GLUT4 into the endosomal system (Figure 2C). The ubiquitin-binding GGA proteins play a role in sorting GLUT4 into GSVs (3,4), and we therefore investigated whether ubiquitination is required to sort GLUT4 into its insulin-sensitive compartment. Differential centrifugation of adipocyte lysates can be used to enrich for GSVs (25,26). It has previously been shown that expression of a dominant negative GGA protein reduces the proportion of GLUT4 found in the 16 000 ×***g*** supernatant of homogenized adipocytes, reflecting the role of GGAs in sorting GLUT4 into GSVs (4). We observed that substantially less of the ubiquitin-resistant HA-G4-7K/R mutant was found in the 16 000 ×***g*** supernatant compared to wild-type GLUT4 (Figure 4B; 31.6± 1% SEM compared to 53.8 ± 0.6% SEM), supporting the hypothesis that the trafficking of this mutant into GSVs is impaired. Distribution of the insulin-responsive aminopeptidase (IRAP), which also occupies GSVs, was unchanged in cells expressing HA-G4-7K/R (Figure 4B).

**Figure 5:**
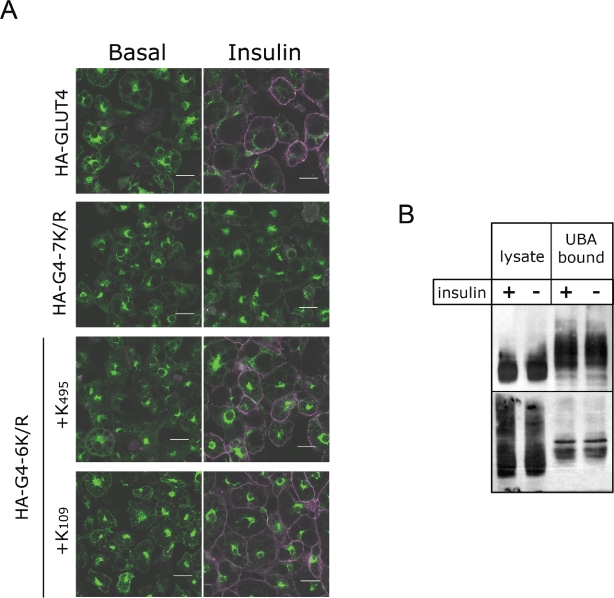
**Insulin-dependent translocation of GLUT4 requires ubiquitin-acceptor sites.** A) 3T3-L1 adipocytes expressing either HA-GLUT4 (WT), HA-G4-7K/R (7K/R), HA-G4-6K/R+K_109_ (+K_109_) or HA-G4-6K/R+K_495_ (+K_495_) were either treated with 100 nm insulin for 20 min (Insulin) or not (Basal), and labeled for surface and internal HA-GLUT4. Surface GLUT4 (labeled with HA antibody without cell permeabilization) is shown in magenta and total GLUT4 (following permeabilization) is shown in green. Scale bar = 20 µm. A total of 91% (±2%) of cells expressing HA-GLUT4 exhibited plasma membrane HA immunoreactivity (rim fluorescence), with similar levels found for cells expressing either of the 2 HA-GLUT4 6K/R constructs (K495: 91 ± 6%, K109: 86 ± 3%). This proportion was significantly less [22% (±4%)] for cells expressing the ubiquitin-resistant HA-GLUT4 7K/R (p < 0.001 compared to cells expressing wild-type HA-GLUT4, unpaired Student's *t*-test). At least 50 cells from three fields of view were counted from three independent experiments, results are expressed ± standard error of the mean. B) Lysate prepared from two 10-cm plates of 3T3-L1 adipocytes that had either been treated with 100 nm insulin for 20 min (+) or not (−) was adjusted to a protein concentration of 5 mg/mL with lysis buffer containing 1 mm NEM. Five hundred microliters of this was incubated with 20 µg of GST-fusion protein harboring the ubiquitin-binding domain of Dsk2p (GST–UBA) immobilized on glutathione-Sepharose at 4°C for 2 h with continual mixing. Following extensive washing, immunoblot analysis was used to estimate the amount of GLUT4 bound to the GST–UBA fusion. Two representative immunoblots are shown. The ‘lysate’ lanes contain 10 µL of a 1 in 50 (upper panel) or a 1 in 2 (lower) dilution of the lysates used in the pull-downs.

**Figure 6:**
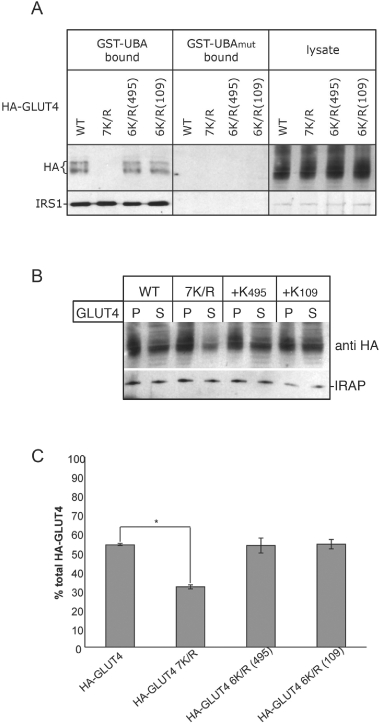
**Further characterization of 6K/R mutant versions of HA-GLUT4.** Cell lysates prepared from 3T3-L1 adipocytes expressing either wild-type HA-GLUT4, HA-G4-7K/R (7K/R), HA-G4-6K/R+K_495_ [6K/R(495)] or HA-G4-6K/R+K_109_ [6K/R(109)] were (A) incubated either GST–UBA or GST–UBA_mut_ immobilized on glutathione-Sepharose as in Figure 3 with the immunoblot being probed for HA to detect the GLUT4 constructs and an antibody that specifically recognizes IRS1 as a positive control, or (B) subjected to 16 000 ×***g*** centrifugation for 20 min (as in Figure 4B). The amount of the HA-tagged versions of GLUT4 and IRAP in the pellet (P) and supernatant (S) was determined by immunoblot analysis. (C) Partitioning of HA-GLUT4 (wild-type and mutants thereof, as above) between pellet and supernatant fractions shown in (B) was compared using image analysis software (ImageJ, NIH) to quantify the optical density of the bands on the blot. The percentage of total HA-GLUT4 in the supernatant fraction is plotted. *n* = 3, *statistically significant difference (p < 0.001, unpaired Student's *t*-test). Error bars ± standard error of the mean.

The data presented in Figure 4 support a model in which ubiquitination of GLUT4 is required to sort the transporter into the compartment(s) from where it can be mobilized to the cell surface in response to insulin. To test this we used a cell surface labeling assay that takes advantage of the HA epitope in the first exofacial loop of HA-GLUT4 to follow the insulin-dependent delivery of wild-type and ubiquitin-resistant GLUT4 to the plasma membrane of adipocytes (Figure 5). Wild-type HA-GLUT4 displayed pronounced translocation to the cell surface in response to acute insulin stimulation, whereas this effect was blunted in the case of the HA-G4-7K/R mutant (Figure 5). To address the possibility that mutation of the seven lysines in GLUT4 might affect its trafficking via a ubiquitin-independent mechanism we reintroduced either Lys_109_ or Lys_495_ in the context of HA-G4-7K/R. Both of these mutants (HA-G4-6K/R+K_109_ and HA-G4-6K/R+K_495_) exhibited robust insulin-dependent translocation to the cell surface similar to that observed for wild-type HA-GLUT4 (Figure 5A). The finding that reintroduction of either of these ubiquitin-acceptor sites into HA-G4-7K/R is sufficient to restore insulin responsiveness (as well as ubiquitination and sorting into a membrane fraction enriched in GSVs/IRVs; Figure 6) demonstrates that there is no strict requirement for a particular lysine residue for the ubiquitin-dependent sorting of GLUT4. This observation means that it will not be possible to map the ubiquitin-acceptor site of GLUT4 using a mutagenesis approach *in vivo*, as it would appear that in the absence of the favored lysine the locale of ubiquitination is shifted, a phenomenon previously reported for other substrates (27). Importantly, the data presented in Figure 5 demonstrate that mutations introduced into the large cytosolic loop of GLUT4 in HA-G4-7K/R do not affect the mutant's ability to traffic in an insulin-dependent manner because of non-specific reasons such as altering the structure of this region of the protein. Consistent with a role of GLUT4 ubiquitination in the sorting of GLUT4 into GSVs rather than in trafficking from GSVs to the plasma membrane in response to insulin, we found that the proportion of GLUT4 that is ubiquitinated is not affected by acute insulin stimulation (Figure 5B).

## Discussion

This study contains the first report that GLUT4 is ubiquitinated. This modification appears to play a crucial role in delivery of GLUT4 to its insulin-sensitive storage compartment. Failure to ubiquitinate GLUT4 results in the loss of insulin responsiveness (Figure 5). Taken in conjunction with the finding that ubiquitination is required to sort GLUT4 into a fraction enriched for GSVs (Figures 4 and 6) and reports demonstrating a role for the ubiquitin-binding GGA proteins in sorting GLUT4 from the TGN into GSVs (3,4), these data support a model whereby ubiquitination of GLUT4 provides the signal for sorting of the transporter into GSVs which are stored in the cytoplasm until an insulin stimulus is received.

Ubiquitin is well documented as a sorting signal through the endosomal system, targeting the delivery of proteins from the TGN, as well as from the cell surface, through the endocytic/multi-vesicular body (MVB) pathway to the lysosome or vacuole (6). Ubiquitin-dependent sorting of proteins from the TGN through this pathway requires the GGA proteins, which serve as clathrin adaptors to recruit ubiquitinated cargo (7). This is exemplified in the nitrogen-regulated sorting of Gap1p in yeast, where the transporter is ubiquitinated under nutrient-rich conditions and delivered to the vacuole (28). This ubiquitin-dependent delivery is facilitated by GGA proteins binding the ubiquitinated transporter at the TGN to sort it into the endosomal system (5). When nutrients are limiting, Gap1p is no longer ubiquitinated, fails to bind the GGAs, and traffics to the cell surface (28).

We originally observed GLUT4 ubiquitination by expressing the transporter heterologously in yeast. We found that, like Gap1p, GLUT4 requires both ubiquitin-acceptor sites and GGA proteins to traffic through the endosomal system. The GGA proteins are required for the insulin-regulated trafficking of GLUT4 in adipocytes, sorting the transporter into its insulin-responsive compartment (3,4) and here we have demonstrated a role for ubiquitination in this same sorting step, underscoring parallels that have been drawn between GLUT4 and Gap1p trafficking (1,29).

While there do appear to be many parallels between the regulated trafficking pathways followed by Gap1p in yeast and GLUT4 in insulin-sensitive cells, there are also clearly many differences. In contrast to ubiquitination of Gap1p, which occurs in response to the stimulus that regulates trafficking of the transporter (28), ubiquitination of GLUT4 occurs under basal conditions rather than in response to insulin (Figures 3 and 5B). This modification is required for the normal insulin-regulated trafficking of GLUT4 in adipocytes. This is consistent with a model in which GGA proteins sort GLUT4 from the TGN into GSVs (3,4). It is important to note that we do not suggest that yeast contain GSVs, but rather that some of the molecular machinery required to sort GLUT4 into GSVs in insulin-sensitive cells are conserved between yeast and adipocytes, namely recognition of ubiquitinated cargo by the GGA proteins for delivery into the endosomal system, and that this is essential for the normal trafficking itinerary of GLUT4.

Interaction between GGA proteins and ubiquitinated cargo targets proteins for lysosomal degradation (6). Consistent with this, GLUT4 is degraded by vacuolar (lysosomal) proteases in yeast in a ubiquitin- and GGA- dependent manner (Figure 2C). How might insulin-sensitive cells use this ubiquitin/GGA pathway to sort GLUT4 into GSVs rather than targeting it to the lysosome? The answer to this question could lie with either the architecture of the ubiquitination bestowed upon GLUT4, or with one of the many deubiquitinating enzymes (DUBs) found in the endosomal system (30). We estimate that approximately 0.1% of GLUT4 is ubiquitinated in adipocytes, indicating that the modification is a transient one (Figure 3B). It may be that GLUT4's ubiquitination is removed after the GGAs have directed it from the TGN, but before it is sorted into MVBs and trafficked to the lysosome. Such reversible ubiquitination is evident in the case of an increasing number of proteins including the epidermal growth factor receptor (31), the *Drosophila* Wnt receptor Frizzled (32) and the β2-adrenergic receptor (33), and further investigations will reveal whether this is also the case for GLUT4.

Ubiquitination has been implicated in the regulated trafficking of a number of other molecules including MHC Class II, aquaporin-2 and the nerve growth factor receptor TrkA (34–36). In these instances ubiquitination appears to control endocytosis, and loss of this modification results in accumulation of the protein at the cell surface. The role of ubiquitination in GLUT4 trafficking is distinct from these examples. The ubiquitin-deficient GLUT4 (G4-7K/R) mutant does not accumulate at the cell surface of adipocytes (Figure 5A). The lack of insulin responsiveness of the G4-7K/R mutant (Figure 5A), taken together with its altered fractionation profile in basal cells (Figure 4B), supports a role for ubiquitination in directing GLUT4 from the TGN/endosomal system into GSVs, a critical step in its insulin-regulated trafficking.

## Materials and Methods

### Reagents

Antibodies against GLUT4 and Vti1p have been described elsewhere (20,37,38). Polyclonal antibodies for Syntaxin16 and IRAP were from Wanjin Hong (IMCB, Singapore) and Paul Pilch (Boston University School of Medicine). Other antibodies were purchased from Synaptic Systems (anti-Syntaxin4 rabbit polyclonal); Upstate (anti-IRS-1); Covance (anti-ubiquitin mouse monoclonal) and Berkeley Antibody Co., Inc. (anti-HA mouse monoclonal 16B12). Yeast strains and plasmids are listed in Tables 1 and 2.

**Table 2:** Plasmids used in this study

Plasmid	Description	Reference
pRM1	Yeast expression plasmid (*CEN*, *LEU2*) encoding HA-tagged ubiquitin from the *CUP1* promoter	This study
pRM2	Yeast expression plasmid (*CEN*, *URA3*) encoding human GLUT4 from the *CUP1* promoter	This study
pRM34	Yeast expression plasmid (*CEN*, *URA3*) encoding human GLUT4 tagged with GFP from the *CUP1* promoter	This study
pRM3	Yeast expression plasmid (*CEN*, *URA3*) encoding human GLUT4 with lysines 109, 242, 245, 261, 264, 266, 495 mutated to arginine residues (G4-7K/R) from the *CUP1* promoter	This study
pGEX-DSK2_UBA_	*E. coli* expression vector encoding GST–UBA (the UBA domain, residues 328–373 fused to the C-terminus of GST, based on pGEX)	(21)
pCAL1	*E. coli* expression vector encoding GST–UBA_mut_ (as above with two point mutations, M342R and F344A in the UBA domain)	This study
pRM55	Retroviral expression vector encoding HA-tagged GLUT4	This study
pRM4	Retroviral expression vector encoding HA-G4-7K/R	This study
pRM35	Retroviral expression vector encoding HA-HA-G4-6K/R+K_109_	This study
pRM36	Retroviral expression vector encoding HA-HA-G4-6K/R+K_495_	This study
pSN222	Yeast expression vector (*CEN*, *LEU2*) encoding HA-tagged Kex2p	(43)

A detailed account of plasmid construction can be found in Appendix S1.

**Table 1:** Yeast strains used in this study

Strain	Genotype	Reference
RPY10	*MATa ura3-52 leu2-3, 112 his4-519 ade6 gal2*	(39)
SF838-9D	*MATa ura3-52 leu2-3, 112 his4-519 ade6 gal2 pep4-3*	(40)
HYY1	*MATa ura3-52 leu2-3, 112 his4-519 ade6 gal2 pep4-3 vps27*Δ*::LEU2*	(15)
SEY6210	*MATa ura3-52 leu2-3, 112 his3*-Δ*200 trp1*-Δ*901 lys2-801 suc2*-Δ9	(41)
MBY004	*MATa ura3-52 leu2-3, 112 his3*-Δ*200 trp1*-Δ*901 lys2-801 suc2*-Δ*9 gga1::HIS5spL gga2::TRP1*	(42)

RPY10, SF838-9D and HYY1 are all congenic, as are SEY6210 and MBY004.

### Cell culture

Yeast were grown in standard minimal media (SD; 0.67% Difco yeast nitrogen base without amino acids, 2% glucose) supplemented with amino acids. Steady-state levels of proteins in yeast cells were assessed through immunoblotting as described (44). 3T3-L1 fibroblasts (American Type Culture Collection) were cultured as described (45). Expression of HA-GLUT4 and mutant versions thereof was achieved as described (45).

### Fluorescence microscopy

Indirect immunofluorescence microscopy in yeast and adipocytes was as previously described (15,17). FM4-64 was used as a marker of the class E compartment in *vps27*Δ cells as described (46). For surface HA-GLUT4 labeling, cells were blocked with 2% BSA, 1 mm glycine in PBS (BSA/gly/PBS) after fixation, followed by incubation with HA antibody and Cy3-conjugated secondary antibody. To assess total levels of HA-GLUT4, cells were subjected to a second fixation prior to permeabilization with 0.1% saponin in BSA/gly/PBS and incubation with HA antibody followed by Alexa_488_-conjugated secondary antibody. Optical sections were analyzed by confocal laser scanning microscopy using a Zeiss LSM PascalExciter fluorescence system. Fluorophores were scanned separately and images overlaid using lsm software.

### Immunoprecipitation of GLUT4

Cells harvested from 500 mL mid-log phase cultures were resuspended in 5 mL YPD-sorb [1% (w/v) yeast extract, 2% (w/v) peptone, 2% (w/v) glucose 50% (v/v) YPD, 1.2 m sorbitol] containing 150 mg yeast lytic enzyme (MP Biomedicals) and 15 µL β-mercaptoethanol. Cells were converted to spheroplasts during a 1-h incubation at 30°C. All subsequent steps were at 4°C. Spheroplasts were layered onto a 10-mL, 1.2-m sucrose cushion and harvested at 3000 ×***g*** for 5 min. Lysis was achieved by brief vortexing following resuspension in 5 mL cold IP-lysis buffer [200 mm sorbitol, 100 mm KOAc, 1% (v/v) Triton X-100, 50 mm KCl, 20 mm PIPEs (pH 6.8), 1 mm*N*-ethylmaleimide (NEM)]. Lysates were cleared by centrifugation (3000 ×***g*** for 5 min), and incubated with 50 µL of a 50% slurry of PrA-agarose (Sigma-Aldrich) for 15 min. PrA-agarose was removed by centrifugation at 10 000 ×***g*** for 5 min. A 10-µL aliquot of cell lysate was reserved for immunoblot analysis, before 1 mL of lysate was added to tubes containing 10 µL anti-GLUT4 antiserum (37). Lysates and antibodies were mixed for 2 h, prior to the addition of 50 µL of a 50% slurry of PrA-agarose (Sigma-Aldrich) and an additional hour of mixing. PrA-agarose with bound antibody and associated proteins was pelleted (5000 ×***g*** for 5 min), washed three times with 1 mL IP-lysis buffer. Bound proteins were eluted in 20 µL Laemmli sample buffer and denatured at 65°C (10 min). PrA-agarose was removed by centrifugation at 10 000 ×***g*** for 5 min. Immunoprecipitated proteins were subjected to immunoblot analyses as indicated.

### GST-fusion pull-downs

GST-fusion protein containing the UBA domain of Dsk2p (residues 328–373; GST–UBA) was purified from *Escherichia coli* (BL21-DE3; Invitrogen) and used in pull-down assays as described (21) from either an adipocyte lysate or yeast cell lysates. Lysates were prepared as described (44,45) with the inclusion of 1 mm NEM in lysis buffers. A version of this protein harboring two point mutations (M342/R and F344/A; numbers refer to positions in full-length Dsk2p; GST–UBA_mut_) was similarly prepared from cells harboring pCAL1.

### Subcellular fractionation of 3T3-L1 adipocytes

Cellular fractions of 3T3-L1 adipocytes enriched in GSVs/IRVs were obtained by a method based on that developed by Li et al. (4). Two 10-cm plates were scraped into 500 µl PBS containing protease inhibitors (Roche complete, EDTA free) and lysed by passage through a 25-gauge needle (10×) and subsequently a 26-gauge needle (2×). The lysate was clarified by centrifugation at 500 ×***g*** for 10 min at 4°C. The resultant supernatant was subjected to further centrifugation at 16 000 ×***g*** for 20 min (4°C). The pellet from this spin was resuspended in PBS containing protease inhibitors. For immunoblot analysis 20 µg (protein) of supernatant and 5 µg (protein) pellet fractions were run on SDS–PAGE gels.

## Supporting Information

**Appendix S1:** Construction of plasmids.
