# Supplementary material for: Insulin-Regulated Trafficking of GLUT4 Requires Ubiquitination
Source: Traffic. 2010 Sep 20;11(11):1445–54. doi: 10.1111/j.1600-0854.2010.01113.x (PMC3152195; doi:10.1111/j.1600-0854.2010.01113.x)
Supplement: Supplementary file 1 [file tra0011-1445-SD1.doc]

**Supporting Information**

**Construction of plasmids made during the course of this study**

pRM1 was constructed by subcloning a *BamI*HI/*Pst*I fragment, containing sequence encoding HA-tagged ubiquitin downstream of the *CUP1* promoter, from Yep112 (1) into pRS315 (2). pRM2 was constructed by amplifying the human GLUT4 ORF from a cDNA kindly provided by Prof. Gwyn Gould (University of Glasgow, U.K.) by PCR using the oligonucleotide primers 5’-GATATTAAGAAAAACAAACTGTACAATCAATCAATCAATCATCACATAAAATGCCGTCGGGCTTCCAACAGATA-3’ and 5’- ATTATAACGTATTAAATAATATGTGAAAAAAGAGGGAGAGTTAGATAGGATCAGTCGTTCTCATCTGGCCCTAA-3’ to generate the GLUT4 ORF flanked by sequences homologous to the 3’ end of the *CUP1* promoter and the proximal end of the 3’UTR of *PHO8* (underlined). This fragment was then used to repair a gapped plasmid encoding Pho8p under *CUP1* regulation (provided by Dr. Rob Piper, University of Iowa, U.S.A.) by homologous recombination. pRM3 was constructed from pRM2 by using site directed mutagenesis to sequentially mutate the codons for the 7 lysine residues (at positions 109, 242, 245, 261, 264, 266 and 495) to codons for arginine residues. pRM4 was similarly constructed, mutating the same codons in the context of the retroviral expression vector pRM55 which was derived from pHA-GLUT4 (3). pRM35 and pRM36 were generated in a similar fashion except the codons encoding K109 and K495 (respectively) were not mutated. pRM34 was constructed by using the oligonucleotide primers 5’-GGAGGTGCGACCCAGCACAGAACTTGAGTATTTAGGGCCAGATGAGAACGACGCATGCATGGGTAAAGGAGAAGAACTTTTC-3’ and 5’- ATTATAACGTATTAAATAATATGTGAAAAAAGAGGGAGAGTTAGATAGGAGCATGCTTACTTGTATAGTTCATCCATGCCATG-3’ to generate a PCR product from pGO36 (4) encoding GFP(S65T) flanked by sequences homologous to the 3’ end of the GLUT4 ORF and the proximal end of the 3’UTR of *PHO8* (underlined). Homolgous recombination was used to insert the GFP-ORF immediately after the last codon of GLUT4 by repairing a version of pRM2 (in which the GLUT4 STOP codon was replaced by an *Sph*I site) that had been linearised by digestion with *Sph*I.

To control for the specificity of interaction between the GST-UBA fusion protein (5) and ubiquitinated proteins two residues in the UBA domain were mutated. The structure of the UBA domain of Dsk2p, present in this fusion protein, in complex with ubiquitin has been solved and demonstrates that Met_342_ of Dsk2p plays an integral role in ubiquitin recognition (6). The methyl group of this residue fits into a hydrophobic pocket on the surface of ubiquitin and its main chain carbonyl group accepts a hydrogen bond from the amide group of Gly_47_ of ubiquitin (6). Gly_47_ of ubiquitin also makes hydrophobic contact with Phe_344_ of Dsk2p and mutation of Phe_344_ of Dsk2p to alanine decreases the proteins affinity for ubiquitin 68 fold (6). pCAL1 was constructed by using site directed mutagenesis to mutate the codons for Met_342_ and Phe_344_ of Dsk2p in the context of the plasmid encoding the GST-UBA fusion protein (5) to codons for arginine and alanine respectively using the oligonucleotide primers 5'-GACAACTAAACGACAGGGGCGCCTTCGATTTCGATAGAAAC-3' and 5'-GTTTCTATCGAAATCGAAGGCGCCCCTGTCGTTTAGTTGTC-3' (mutagenic codons are underlined).

**References for Supporting information**

1. Hochstrasser M, Ellison MJ, Chau V, Varshavsky A. The short-lived MAT alpha 2 transcriptional regulator is ubiquitinated in vivo. Proc Natl Acad Sci U S A 1991;88:4606-4610.

2. Sikorski RS, Hieter P. A system of shuttle vectors and yeast host strains designed for efficient manipulation of DNA in Saccharomyces cerevisiae. Genetics 1989;122:19-27.

3. Shewan AM, Marsh BJ, Melvin DR, Martin S, Gould GW, James DE. The cytosolic C-terminus of the glucose transporter GLUT4 contains an acidic cluster endosomal targeting motif distal to the dileucine signal. Biochem J 2000;350:99-107.

4. Odorizzi G, Katzmann DJ, Babst M, Audhya A, Emr SD. Bro1 is an endosome-associated protein that functions in the MVB pathway in Saccharomyces cerevisiae. J Cell Sci 2003;116:1893-1903.

5. Funakoshi M, Sasaki T, Nishimoto T, Kobayashi H. Budding yeast Dsk2p is a polyubiquitin-binding protein that can interact with the proteasome. Proc Natl Acad Sci U S A 2002;99:745-750.

6. Ohno A, Jee J, Fujiwara K, Tenno T, Goda N, Tochio H, Kobayashi H, Hiroaki H, Shirakawa M. Structure of the UBA domain of Dsk2p in complex with ubiquitin molecular determinants for ubiquitin recognition. Structure 2005;13:521-532.
